# Beyond satisfaction: Using the Dynamics of Care assessment to better understand patients' experiences in care

**DOI:** 10.1186/1477-7525-6-20

**Published:** 2008-03-10

**Authors:** Bruce Rapkin, Elisa Weiss, Rosy Chhabra, Laura Ryniker, Shilpa Patel, Jason Carness, Roberto Adsuar, Wendy Kahalas, Carol DeLeMarter, Ira Feldman, Judy DeLorenzo, Ellen Tanner

**Affiliations:** 1 Department of Psychiatry and Behavioral Sciences, Memorial Sloan-Kettering Cancer Center, New York, USA; 2 AIDS Institute, New York State Department of Health, Albany, USA; 3 Department of Pediatrics, Albert Einstein College of Medicine, Bronx, USA

## Abstract

**Background:**

Patient perceptions of and satisfaction with care have become important indicators of the quality of services and the relationship of services to treatment outcomes. However, assessment of these indicators continues to be plagued by measurement problems, particularly the lack of variance in satisfaction data. In this article, we present a new approach to better capture patient perceptions of experiences in care, the Dynamics of Care (DoC) assessment. It is an in-depth approach to defining and assessing patients' perspectives at different junctures in care, including their decisions about whether and where to seek care, the barriers encountered, and the treatments and services received.

**Methods:**

The purpose of this article is to describe, validate, and discuss the benefits and limitations of the DoC, which was administered as part of a longitudinal study to evaluate the New York State HIV Special Needs Plan (SNP), a Medicaid managed care model for people living with HIV/AIDS. Data are from 426 study respondents across two time points.

**Results:**

The results demonstrate the validity and value of the DoC. Help seeking decisions and satisfaction with care appear to be situation-specific, rather than person-specific. However, barriers to care appear to be more cross-situational for respondents, and may be associated with clients' living situations or care arrangements. Inventories in this assessment that were designed to identify potential deterrents to help seeking and difficulties encountered in care demonstrated clear principal component structures, and helped to explain satisfaction with care. The problem resolution index was found to be independent from satisfaction with care and the data were more normally distributed. DoC data were also associated with subsequent utilization and change in quality of life.

**Conclusion:**

The DoC was designed to be a flexible, integrated measure to determine individuals' salient service needs, help seeking and experiences in care. One of the many strengths of the assessment is its focus on specific problems in context, thus providing a more sensitive and informative way to understand processes in care from the patient's perspective. This approach can be used to direct new programs and resources to the patients and situations that require them.

## Background

There is mounting evidence that variations in perceived quality of health care among people with HIV/AIDS affect patient behavior, especially adherence to medication regimens and other physician recommendations, as well as health outcomes [1-9]. As a result, client satisfaction has become the most important direct feedback to providers on the quality of services and the relationship of services to treatment outcomes for many health care organizations [2]. Cherin et al. (2001) note that "patient satisfaction with services, as a cornerstone of quality care, has emerged as an important focus of managed care organizations (MCOs) over the last decade. Member dissatisfaction with services has been demonstrated to have a direct impact on plan disenrollment" (p. 105).

Funding agencies have also placed an emphasis on clients' perceptions of care. The Health Resources and Services Administration (HRSA), in providing guidance to its Ryan White CARE Act grantees, has recommended that service planners and providers pay close attention to client satisfaction as they develop and implement their service delivery systems [10]. The emphasis in the private and public sectors on perceived quality of care has led health and managed care industry research organizations (e.g., Group Health Association of America, RAND Corporation, The Measurement Group) to invest considerable time and expense in developing patient care satisfaction instruments tailored to managed care organizations. These instruments tend to define satisfaction in this context as a patient's perception of the quality of physicians, access to services, communication with providers and administrative staff, and of the success of their treatment [3].

Despite widespread consensus that perceived quality of care, and patient satisfaction in particular, is important to measure, and despite much investment in instrument development, the assessment of patient satisfaction has been and continues to be plagued by critical measurement issues [11]. There is substantial debate about whether patient satisfaction can be measured reproducibly and meaningfully [12-16]. Ware's work (1978) highlights the bias in patient satisfaction questionnaires due to acquiescent response set (ARS), a tendency to agree with statements of opinion regardless of content. More than twenty years after Ware's seminal article discussed this problem, researchers are still encountering negatively skewed distributions in satisfaction measures [3,15]. Moreover, Ware (1978), in his review, found that biases were greatest for groups reporting lower educational attainment or less income, which makes this problem of paramount concern in the study of quality of care for many segments of the HIV/AIDS-affected population. The literature also suggests that when we look at satisfaction with managed care programs among patients with HIV/AIDS, men consistently rate such programs higher than women [3], and that attitudes about what aspects of care delivery are most important vary by gender and race/ethnicity [4].

Several studies suggest that health status per se, rather than degree of improvement in health status due to medical care, also influences satisfaction ratings; there is some evidence that healthier patients tend to report greater satisfaction with health care [11,14,17]. Statistical adjustments can be made to deal with sociodemographic or health status confounds that may occur when trying to predict patient behaviors such as adherence. However, if the purpose is quality improvement, adjustment hides what may be important problems and hinders the development of innovative solutions for different patient groups [14]. In HIV care, studies suggest that it may be particularly important to understand how different patient groups experience care and what people with different backgrounds value, so that interventions and changes in care delivery practices can be appropriately tailored [2,4,7,12,18].

Given the pitfalls of satisfaction measurement and its debatable value for quality improvement, there has been a call for new approaches to the assessment of patient perceptions of care. A number of researchers have emphasized the need for increased attention to narrative, specifically patients' detailed accounts of what went well and what did not, as well as reports about what did or did not happen when patients received care, what they experienced in light of what they value, and whether needs have been met [4,5,8,14,19]. Cleary and Edgman-Levitan (1997) note that this kind of information tends to better reflect the quality of care and is also more "interpretable and actionable for quality improvement purposes" (p. 1608). This is particularly the case when looking at the quality of care for patients with complex needs and many contacts with providers, since these patients are likely to perceive different aspects of care differently (e.g., a patient with HIV may be satisfied with help with drug use but not with the support provided for medication adherence). Cleary and Edgman-Levitan (1997) have shown that people with complicated conditions do not describe their experiences in terms of single visits with one provider, but in terms of episodes of care, and these authors call for developing ways of collecting quality information for entire episodes of care (p.1611).

These concerns led us to develop a novel approach to the assessment of health service use and quality, which we refer to as the "Dynamics of Care" assessment. The purpose of this article is to describe, validate, and discuss the benefits and limitations of the Dynamics of Care assessment, which was developed for a longitudinal evaluation study called "Choices in Care." The study was designed to evaluate New York State's Special Needs Plan – a comprehensive care model created for people with HIV and their children that was developed as an alternative to both Medicaid fee-for-service (FFS) and to Medicaid managed care [20]. The larger Choices in Care evaluation examines access to care and quality of care as well as patient reported outcomes such as treatment adherence, risk behaviors, and quality of life. In this paper, we focused solely on the validation of the Dynamics of Care assessment.

## Methods

### Sample

Individuals were eligible to participate in the Choices in Care study if they enrolled in one of the Special Needs Plans (SNPs) in the prior three months, or if they received care through the traditional Medicaid FFS system. Enrollment in a SNP is voluntary, and the majority of people with Medicaid in New York City who are HIV+ are still receiving care through the FFS model. Thus, we oversampled SNPs enrollees to facilitate comparison with FFS. New SNP enrollees and FFS recipients were eligible for the study if they were 18 years of age or older, spoke either English or Spanish, resided in New York City, had the cognitive capacity to complete the informed consent and a 1 hour interview, and were not incarcerated at the time of the recruitment call.

The Special Needs Plan model began in 2003. To date, the three active plans have an enrollment of over 2000 individuals. Study recruitment began in September, 2003, following approval from the Institutional Review Boards at Memorial Sloan-Kettering Cancer Center and the New York State AIDS Institute; recruitment concluded in January, 2007. We recruited new SNP enrollees within 45–90 days of their enrollment into one of the three plans, in order to get baseline information about their health status upon entering the plan, before they had an opportunity to make use of SNP services. New SNP Enrollees were identified from a roster of names provided monthly by the New York State Department of Health (NYSDOH) AIDS Institute. It is important to note that upon enrolling in a SNP, individuals were informed that they would be contacted for plan evaluation purposes. While all individuals had the opportunity to take part in the study, only a percentage were reached by phone and agreed to participate during the eligible timeframe (up to 90 days post enrollment). The comparison group of fee-for-service recipients was recruited concurrently as a convenience sample; they self-selected for the study by responding to flyers posted at Designated AIDS Centers, at community based organizations, and Adult Day Health Care programs. The majority of these recruitment sites provide care to HIV+ individuals under the fee-for-service model and to people enrolled in one of the Special Needs Plans. For privacy reasons, it was not feasible to obtain a list of all HIV+ fee-for-service recipients from which to recruit.

The purpose of the Choices in Care evaluation is to examine access to care, perceived quality of life, member satisfaction and patient reported outcomes among HIV+ adult medicaid recipients. Since the study's inception, 306 SNP enrollees and 322 FFS recipients have been recruited, for a total of 628 subjects. The study refusal rate among known eligible SNP enrollees was 12%. The majority of analyses reported here are based on data from 426 respondents who have completed the first and second (of five) study interviews.

### Procedures

Study participants were interviewed five times, at three month intervals, in order to compare patient needs, access to care, quality of care, and patient outcomes across the SNP and FFS samples, and within the SNP sample across the three plans. The present validation study draws on data from the baseline and three-month interviews. The informed consent process occurred at the time of the baseline interview. Each interview took 30–45 minutes to complete and was conducted either in person at the study offices in New York City or on the phone, whichever was more desirable for the participant. Interviews were conducted in English or Spanish, depending on the participant's preference. In addition to transportation reimbursement, participants received $25 for the first interview, $30 for the second, $35 for the third, $40 for the fourth and $45 for completing the fifth.

### Measures

Information about personal characteristics, including self-identified gender, age, race/ethnicity, whether the respondent's primary language is English or not, and education level (high school graduate or not), was obtained in the baseline interview. The Choices in Care study was designed to examine impact of care on patient reported outcomes. Thus, outcome measures were collected at baseline and again at six months, including health status, treatment adherence, risk behaviors, and quality of life. The main Dynamics of Care assessment occurred at the intervening three month interview, with brief follow-up questions occurring during the six month interview, including questions about the current status and resolution of events in care identified at the three month time point (see discussion of measure below).

#### The Dynamics of Care assessment

The Dynamics of Care assessment was designed to capture patients' experiences of care in detail, for the purpose of evaluation and quality improvement. It is an in-depth approach to defining and assessing patients' perspectives at different junctures during an episode of care, including their decision whether and where to seek care, the barriers they encountered, and the treatments and services they received. The Dynamics of Care is an interviewer administered assessment that begins by asking respondents whether they experienced problems in the last three months in nine specific areas ("trigger events") that the Special Needs Plan was designed to address. These areas include: 1) Adherence to Medical Instructions; 2) Medical Problems; 3) Specialty & Inpatient Hospital Care; 4) Preventive Health Care & Screening; 5) Sexual Risk Behavior; 6) Family Planning; 7) Psychological Symptoms; 8) Substance Use; and 9) Life Circumstances & Demands. This first part of the measure, the Events in Care Screening Questionnaire (ECSQ) contains 54 yes/no items and serves to identify the areas of recent concern to each patient [21]. Respondents are also asked to rate satisfaction with the services that are available to them in each of these nine areas on a scale from 0 (Completely Dissatisfied) to 10 (Completely Satisfied), regardless of whether they identify recent events in care in a given area. Satisfaction ratings are used to identify areas for further discussion and probing.

The main part of the Dynamics of Care interview explores respondent experiences, with respect to three selected events in care. The interview is structured with skip patterns to probe relevant questions depending upon how far individuals have progressed in regard to seeking and obtaining care. For each of the three trigger events, respondents are asked to provide a brief narrative description of the concern. They then delineate whether or not they sought care and what factors influenced that decision, including provider recommendations. Among those who were or are actively seeking care, we ask about any barriers and delays that they have encountered. For those who have already started receiving services it is determined which providers they have seen; respondents are then asked about experiences in care including the outcome of referrals, coordination of care, and communication with providers. Respondents are also asked to rate satisfaction with help received from the main provider involved with their care pertaining to each event. In addition, overall experience of problem resolution is explored including whether or not they are seeking or receiving care, or trying to handle a problem on their own.

If a participant mentions problems in more than three of the nine areas, three events are selected for further probing based on the area of greatest concern to the patient, the area that the patient rated *highest *in terms of satisfaction with care available, and the area the patient rated *lowest *in terms of satisfaction with care available. If more than one area receives an equally high or an equally low satisfaction rating, interviewers were instructed to select area(s) that tended to be identified as concerns less frequently, in our initial piloting of the ECSQ measure (i.e., concerns related to sexual risk, family planning, and substance use). This procedure effectively oversampled infrequent concerns, which made it possible to conduct analyses related to less frequent areas of concern. In sum, the Dynamics of Care assessment guides respondents to identify key events related to their care, and to describe what has transpired regarding that event up to that point. By tracing specific episodes in this way, it is possible to understand discrete circumstances and cause-effect associations based upon each individual's immediate experiences. In essence, this approach trades the breadth and lack of specificity inherent in global satisfaction ratings for a high degree of precision in the assessment of narrowly framed but highly salient experiences. As we shall detail in description of analyses below, the Dynamics of Care measure may be examined with respect to specific areas of care or summarized across areas to create a variety of different summary variables.

#### Health care

• *Number of health care providers and composition of health care team*: Respondents were asked to identify all of the health care providers that were part of their current health care team or whom they had seen in the six months preceding the baseline interview for care.

• *Plan enrollment status*: Respondents were classified as a member of one of the NYSDOH Special Needs Plans, or as a Medicaid fee-for-service (FFS) recipient.

#### Health history and health status

• *Years since HIV diagnosis*: Respondents were asked the month and year when they first tested positive for HIV.

• *CD4 Count*: Measured as less than or equal to 200, or greater than 200, based on respondent report.

• *Undetectable viral load: *Determined by a response of "undetectable" to the question "Do you know or remember the results of your last viral load count?"

• *HIV-related diagnoses*:

∘ *Total number *is based on respondents' indication of whether they had ever been diagnosed with any of 11 different opportunistic infections, disorders and malignancies (e.g., pneumonia PCP, Kaposi sarcoma,).

∘ *Number of conditions for which the respondent was not receiving treatment *was determined by asking respondents to indicate whether the condition had been completely treated, whether they were being treated at that time, or were never treated.

• *Number of symptoms: *Respondents were asked if they had any of 11 different psychological and physical symptoms in the past four weeks. Examples of symptoms include trouble with thinking, concentrating, or memory; fever, chills, sweats.

• *Physical and psychological quality of life composites from the MOS-SF36: *This widely used 36-item measure of health related quality of life and functional status yields physical and psychological quality of life composites, each scored from 0–100 with higher scores indicating greater well-being [22].

#### Risk history and current risk behaviors

The respondents were asked their history and current activity (within the last 3 months) in a variety of risk behaviors including:

• *History of and current hard drug (heroin, crack, other illicit drug, and injected drugs) use*

• *Sexual history- *having a sexual partner who is an injection drug user; having a sexual partner who is HIV seronegative; whether or not the respondent was ever a sex worker; and unprotected sex behavior

• *Criminal justice involvement*

• *Tobacco use*

• *Excessive alcohol use*

#### Personal characteristics

Variables include: self-identified gender; age; race/ethnicity; whether the respondent's primary language is English or not; education level (high school graduate or not); number of sources of income (0–9); monthly gross income from all sources; housing stability (whether or not the respondent lives in a place of his/her own); whether the respondent self-identified as gay or bisexual; whether the respondent had a spouse or partner; whether the respondent was living with a partner; number of other adults and minor children in the household; and whether the respondent was living with another seropositive person.

### Analysis

Analyses were based on data from 426 respondents who completed the first two study interviews. All data were analyzed using SPSS V12.0 for Windows. In the results section that follows, we first present the characteristics of the sample, including demographic information, health status, health care utilization, and risk behavior. We next describe our identification and sampling of Events in Care for further assessment in the Dynamics of Care. Finally, we present a series of analyses of the psychometric and response properties of each section in the Dynamics of Care, including stage of help-seeking, barriers and delays encountered while seeking help, and perceived quality of interaction with main providers. For each section of the measure, we give the item response rates and intraclass correlations across respondents' specific areas of care. We also provide data on inter-item reliability and the principal component structure of summary scores. Lastly, we determine whether Dynamics of Care measures were related to demographic and health-related measures through a series of multiple regression analyses.

## Results

### Sample characteristics

Detailed demographic characteristics are outlined in Table 1. With respect to respondents' involvement with the health care system, 33% of respondents were enrolled in one of the Special Needs Plans; 77% received care through traditional Fee-for-Service. Nearly all had a primary medical care provider. Most people in the sample reported having at least one case manager (82%) and at least one medical specialist (76%) within the six months preceding our baseline interview. Forty-one percent indicated that they have seen a dentist in the past six months; 66% have seen a therapist, psychologist, or psychiatrist; 24% had seen a social worker or other patient supporter, such as a patient advocate; and 7% had a physical therapist, occupational therapist, or rehabilitation therapist on their health care team. Thirty-three percent of respondents had seen a dietitian, nutritionist, or exercise specialist in the past six months.

**Table 1:** Sample Characteristics

	Total Sample N = 426
*Gender*	
Female	49.5%
*Average age*	46 years
*Race/Ethnicity*	
Black	61%
Hispanic	39%
*Primary language*	
Spanish	18%
*Education level*	
High school diploma or GED	64%
Bachelor's or higher degree	8.4%
*Sexual orientation*	
Gay or Bisexual	26%
*Have spouse or partner*	26%
*Living situation*	
Live with spouse or partner	21%
Live with adult other than spouse or partner	35%
Live with minor children	19%
Live with an individual who is seropositive	16%
*Unstable housing ( *Does not have a place of their own)	10%
*Reported working*	14%
*Involved in the criminal justice system (at some time)*	48%
*Average number of years diagnosed*	9.7 years
*CD4 Count greater than 200*	83%
*Undetectable viral load*	46%
*Average number of opportunistic infections*	2.3
Had 2 or 3 conditions	38%
Had 4 or more conditions	24%
Reported never receiving treatment for one or more conditions	27%
*Average number of physical and psychological symptoms*	4.27
*Average score on the MOS physical composite scale*	63.7 (out of 100)
*Average score on the MOS psychological composite scale*	64.3 (out of 100)

With respect to risk behaviors, 75% of the sample has a history of crack, heroin, or other injection drug use; 23% had used one or more of these drugs in the past three months. Fifty-nine percent had sex with someone who injects drugs, and 18% have traded sex for money, drugs, or other needs. Nineteen percent of respondents reported having unprotected sex in the past three months, and 7% had unprotected sex with someone who is seronegative. Twenty-one percent had smoked one or more packs of cigarettes a day in the past three months, and 10% reported that they had too much alcohol in the past three months.

### Events in Care

The first part of the Dynamics of Care assessment entails identifying and prioritizing events of interest to the respondent. As noted above, we developed the ECSQ, a face-valid measure including 54 items that probe needs in nine areas of concern. In each area, we asked whether people living with HIV/AIDS (PWHAs), had specific problems or concerns in the past three months, whether they needed additional information, and whether their providers had raised concerns. Detailed results of the validation of the ECSQ are presented in a separate paper [21]. Responses to the Events in Care measure are detailed in Table 2. For each area, we indicate the proportion of PWHAs that endorsed each item as well as the proportion that endorsed any item in that category. The most prevalent areas of concern included Medical Problems (66%), Specialty and Inpatient Hospital Care (56%), Psychological Symptoms (54%), and Preventive Health Care & Screening (50%). Endorsement frequencies varied substantially across individual items. The most common specific concerns included a Psychological Symptom, "bothered by sadness or depression" (48%) and a Medical Problem Symptom, "becoming more tired and having less energy" (47%). Across all nine areas, 50% of PWHAs indicated that they needed additional information in at least one area and 48% stated that a provider had raised some concern regarding their care. On average, PWHAs endorsed items in 3.7 areas of concern (sd = 2.3), or a total of 1568 events in care.

**Table 2:** Rates of trigger concerns over the past three months, by area and item (N = 428)

**Adherence to Medical Instructions**	**43%**
Difficulty taking medications, like forgetting, being late, prescriptions	21%
Difficulty following medical advice	8%
Any side effects that interfered with activities or made you feel worse	29%
Questions about treatment or lab tests	14%
Any provider expressed concern about adherence or make treatment changes	16%

**Medical Problems and Symptoms**	**66%**

Any new symptoms or health problems	28%
Pain or symptom gets noticeably worse	27%
More tired or have less energy	47%
Any medical problem interfered with activities or ability to care for yourself	23%
Any questions about health symptoms	29%
Seek treatment at an emergency room	20%
Provider expressed concerns about your symptoms, pain or energy level	21%

**Specialty & Inpatient Hospital Care**	**56%**

Required a medical specialist	23%
Any dental problems	23%
Any problems with vision	29%
Any elective surgery or procedure	8%
In hospital or institution (medical)	9%
Provider suggested a medical specialist	18%

**General Wellness and Prevention**	**50%**

Want check-up or screening for blood pressure, diabetes, cancer, etc.	23%
Change habits: diet, smoking, exercise	38%
Have any questions about staying healthy to discuss with provider	29%
Provider said you were overdue for any procedure, screening or other tests	11%

**Substance Use**	**11%**

Increased use of alcohol, marijuana, heroin, cocaine or drugs	7%
Your concerns about alcohol, drugs	9%
Provider concerned re: alcohol, drugs	8%

**Sexual Health and Risk Behavior**	**31%**

Find better ways to prevent sexual transmission of HIV or other infections	27%
Difficulty practicing safer sex	6%
Difficulty talking about HIV	4%
Want to discuss sexual risk and protection with provider	8%
Provider expressed concern about sexual risk or not protecting yourself	9%

**Family Planning and Birth Control**	**14%**

Want better ways to prevent pregnancy	7%
Made changes to birth control choices	2%
Considering changes to birth control	2%
Thinking about having a baby	9%
Want to discuss birth control or family planning with provider	4%
Provider had concerns, changes to birth control or family planning decisions	3%

**Psychiatric Symptoms**	**54%**

Bothered by sadness or depression	48%
Felt panicky, anxious or out of control	32%
Confusion or difficulty concentrating interfered with activities or self-care	29%
Thoughts of harming yourself or others	6%
In hospital or institution (psychiatric)	2%
Questions about coping with feelings or changes in thinking or memory	22%
Provider suggested psychological help	20%

**Dealing with Life Circumstances**	**41%**

Medicaid coverage, loss of income, benefits, housing, or legal problems	22%
Needs of others (like children, family, partner) interfere with self-care	4%
Increased responsibilities at home	16%
Caring for someone sick or disabled	12%
Questions about how to handle problems with money, housing, family	11%
Provider discussed problems related to living situation and care giving	11%

### Selection of concerns for further assessment

Due to practical limitations of time, we decided to ask respondents the Dynamics of Care questions in only three of their identified areas of concern. As mentioned above, in order to select a representative sample of clients' concerns for further discussion in the Dynamics of Care assessment, we first asked respondents to rate their satisfaction with services available to them in each area, in order to select areas in which they were most and least satisfied. Then, we asked them to indicate which area represented their "biggest concern or need." These ratings allowed us to follow-up on clients' most salient concerns, as well as additional areas of concern that varied in terms of level of satisfaction with services. When several different areas received a respondent's highest or lowest rating, interviewers were instructed to select one of the areas that were mentioned less frequently in our pilot data, specifically, Family Planning, Sexual Risk Behavior or Substance Use. In other words, we designed the process to over-sample areas that tended to be mentioned less frequently. Table 3 summarizes the results of this process.

**Table 3:** Rates of Selection of Areas to Probe in Dynamics of Care Interviews

	1	2	3	4	5	6
	Chosen as Most Important	Chosen as Most Satisfied	% Chosen as 2^nd ^Area to Probe	Chosen as Least Satisfied	% Chosen as 3^rd ^Area to Probe	% of All Events Chosen

**Adherence to Medical Instructions**	10%	52%	63%	29%	83%	15%
**Medical Problems**	21%	41%	17%	27%	50%	15%
**Specialty & Inpatient Hospital Care**	13%	46%	48%	32%	72%	16%
**Preventive Health Care & Screening**	7%	48%	30%	34%	58%	10%
**Substance Use**	5%	31%	87%	15%	86%	4%
**Sexual Risk Behavior**	4%	51%	84%	22%	79%	10%
**Family Planning**	3%	43%	100%	11%	100%	5%
**Psychological Symptoms**	16%	40%	26%	30%	56%	13%
**Life Circumstances & Demands**	20%	32%	30%	25%	66%	13%

Explanation of Columns

1. Proportion of time each area was identified as the most important concern to discuss.

2. Proportion of time area was identified as one of the areas with most satisfactory care.

3. Proportion of time area was chosen when it was eligible for the second slot.

4. Proportion of time area was identified as one of the areas with least satisfactory care.

5. Proportion of time area was chosen when it was eligible for the third slot.

6. Proportion of time area was chosen for discussion in any slot out of all selected areas.

Note: Up to three areas selected for discussion included PWHAs' biggest need or concern and areas of greatest and least satisfaction with available services. Since levels of satisfaction could be tied, interviewers were instructed to break ties by selecting areas that were mentioned less frequently in pilot work (family planning, sex risk and substance use. The goal was to balance the number of times we probed each area. As Column 6 demonstrates, we were successful in over-sampling these areas.

Life Circumstances & Demands (20%), Medical Problems (21%), and Psychological Symptoms (16%) were identified most often as respondents' biggest areas of concern, in contrast with Substance Use (5%), Sexual Risk Behavior (4%), and Family Planning (3%) (see Table 3, Column 1). Areas identified as being of greatest concern were probed further in the Dynamics of Care assessment.

Among those people who had indicated concerns in a given area, areas most likely to be given the highest satisfaction ratings included Adherence to Medical Instructions (52%), Sexual Risk Behavior (51%), Preventive Health Care & Screening (48%) and Specialty and Inpatient Hospital Care (46%) (see Table 3, Column 2). Respondents were least likely to select the highest satisfaction rating for Services related to Substance Use (31%) and to Life Circumstances & Demands (32%). Based on our priorities for sampling, selection of events in care for subsequent probing ranged from 100% for Family Planning to 17% for Medical Problems (see Table 3, Column 3).

Areas most likely to receive clients' lowest satisfaction ratings included Preventive Health Care and Screening (34%), Specialty and Inpatient Hospital Care (32%), and Psychological Symptoms (30%). Areas least likely to receive lowest ratings included Family Planning (11%) and Substance Use (15%) (see Table 3, Column 4). Since low satisfaction ratings were less prevalent than high, a higher proportion of these events were probed, ranging from 50% of medical problems to 100% of family planning concerns (See Table 3, Column 5).

Overall, the strategy of selecting respondents' greatest concern, considering other areas according to level of satisfaction, and over-sampling less common concerns yielded good representation across the nine areas under consideration. We were able to probe 969 events in care, which represents 62% out of a total of 1568 events indicated by the ECSQ. The distribution of these events ranged from 4% in the area of Substance Use to 16% in the area of Specialty and Inpatient Hospital Care (see Table 3).

### Overview of analysis of the Dynamics of Care

We conducted a series of analyses on the Dynamics of Care assessment to better understand the psychometric and response properties of each section. The Dynamics of Care assessment was designed to be used in several different ways. The focus on specific events in care makes it possible to examine similar issues across different clients and settings (for example, comparing all episodes in the area of adherence). It is also possible to combine like items across areas of assessment, to derive idiographic (that is, individually-defined) measures of processes in care that are comparable across individuals, despite differences in events in care. For example, it is reasonable to examine individuals' average satisfaction across providers. Similarly, we can indicate whether clients ever encountered a particular barrier in seeking care, irrespective of the specific events in care under consideration.

For the present paper, we have focused analysis on summary ratings derived from the dynamics measurements. This provided an opportunity to consider how baseline individual differences in health care, health status, and personal characteristics related to experiences in care. In order to present analyses in a cogent fashion, we will focus on each section of the interview in sequence: Stage of Help-Seeking, Barriers and Delays Encountered while Seeking Help, Perceived Quality of Interaction with Main Providers and Problem Resolution. This is necessary because sample size changes from section to section (e.g., we only discuss barriers encountered while help-seeking among those respondents who actually sought help). This sequential approach also conveys how the assessment can highlight and focus on different junctures in care.

For each section of the measure, we present information on item response rates, as well as item homogeneity (intraclass correlations) across respondents' sampled areas of care. We also provide information on data reduction procedures, including inter-item reliability (Cronbach's alpha coefficient) and principal component structure of various summary scores. These results are presented in the narrative or in separate tables, below. Finally, we describe results of a series of multiple regression analyses to determine whether Dynamics of Care measures were related to demographic and health-related measures in a coherent fashion. In order to filter the large number of predictor measures and focus in on the strongest and most reliable associations with each dynamics variable within the different sections, we conducted separate regression analyses to identify the strongest predictors from each set of measures, and then considered only those predictors in a final, overall analysis. Results of all regression analyses are presented in similar format, summarized in Tables 4,6,7,9. We will refer back to these tables throughout the presentation of results. We also examined zero-order correlations to identify possible instances of multicollinearity, and will comment on those findings in the description of each analysis.

**Table 4:** Regression Analyses – Stage of Help-Seeking

**% Already Receiving Help**		0.13	p < .001
Dealing with Life Circumstances (-0.21)	Dental Care (0.16)	Recent Unprotected Sex (-0.14)	Spanish Speaking (0.11)
	Number of Case Managers (-0.08)		Any Employment (0.10)
	Psychiatric or Mental Health Care (0.14)		Criminal Justice Involvement (-0.13)
	Problems Recognized by a Provider (0.10)		

**% Seeking Help**		0.12	p < .001

Dealing with Life Circumstances (0.18)	Dental Care (-0.14)	Physical QOL (-0.10)	Spanish Speaking (-0.09)
Specialty and Inpatient Care (0.15)	Number of Case Managers (0.12)	Recent Unprotected Sex (0.15)	Any Employment (-0.11)
			Lives with Other Adults (0.13)

**% Considering Help**		0.07	p < .001

Substance Use (0.15)	Problems Recognized by a Provider (-0.08)	CD4 Count Greater than 200 (0.09)	Age (-0.11)
General Wellness and Prevention (0.11)		Alcohol Use (0.08)	Lives with Another HIV+ Person (-0.09)

**% Would Not Consider Help**		0.09	p < .001

Specialty and Inpatient Care (-0.13)	Number of Specialists (-0.12)	Number of Symptoms (-0.09)	Latino Identity (-0.16)
	Dental Care (-0.11)		High School Graduate (-0.12)
	Number of Case Managers (-0.08)		Criminal Justice Involvement (0.11)
	Special Needs Plan Enrollee (-0.10)		

### Stage of help-seeking and factors that influenced help-seeking decisions

The first section of the Dynamics of Care interview assesses whether and when individuals started to seek help for each sampled event in care. To be selected, these events had to have been a concern at some point during the three months prior to the interview; however, the onset of these concerns could have occurred earlier. Thus, of the 969 events sampled for probing, 16% had emerged as problems in the last month and an additional 28% occurred in the past three months. Of the remaining events, 13% emerged within the past six months, 14% within the past year, and 29% had been problems for a year or more. Respondents had already started to receive help for 52% of the sampled events, were actively seeking help for 16%, and were considering seeking help for 21%. For the remaining 11% of events, respondents said that they would not consider seeking professional help. Across 387 individuals (of a possible 428) who identified at least one event in care, 80% were already receiving help for at least one of their sampled concerns, 34% were seeking help for at least one concern, 41% were contemplating help seeking in some area, and 23% mentioned at least one area in which they would not consider seeking professional help. Among 262 individuals for whom three events were sampled, intraclass correlations (ICC) in help seeking status indicators were relatively low across areas of care, ranging from .02 to .09. (ICC can be interpreted as a correlation between the measure and some indicator of class membership – in this case that events were all reported by the same individual). The timing of the onset of individuals' different concerns also had a moderate ICC = .23. These results suggest that the interview succeeded in eliciting discussion of individual problems and concerns that were distinct from one another.

In order to study differences in help seeking, we created composite scores reflecting the proportion of events for which an individual was receiving, seeking, contemplating or avoiding help. Correlates of these composites are presented in Table 4. Help seeking status was strongly associated with differences in areas of concern. People dealing with life circumstances were less likely to be receiving assistance but more likely to be seeking assistance. Alternatively, people concerned about specialty care were more likely to be seeking assistance, and, as would be expected, were less likely to say that they would not consider professional help. People with substance use and wellness concerns were more likely to say that they were considering initiation of care but had not yet done so.

Help seeking was also associated with presence of providers. As expected, receipt of help was generally associated with provider recognition of problems and presence of different types of providers. However, people seeking help tended to report greater involvement with case managers, especially compared to people who were already connected to services. Help seeking was also associated with individual health status. People who reported poorer physical quality of life at baseline were more likely to be seeking care, while those with fewer symptoms were more likely to say that they would not consider professional help. Alternatively, recent sexual risk behavior and alcohol use were associated with seeking and contemplating care, respectively. Help seeking was also related to indicators of socioeconomic status, with employed individuals more engaged in care, in contrast with those who were recently involved in the criminal justice system or who had not completed high school. Ethnic differences also emerged. Spanish-speaking Hispanics were more likely to have been receiving help, but respondents who identified as Hispanic reported being less likely to not consider seeking help. Active help seeking was also associated with living with other adults and particularly other HIV+ individuals. Younger respondents were also more likely to be considering help.

In order to better understand help seeking, the Dynamics of Care assessment includes a series of nine items that ask about beliefs and preferences that might deter help seeking. These items and their orthogonal varimax principal component structure are presented in Table 5. This analysis was conducted on events (not individuals), so that it would be possible to directly compare factor scores generated for different areas of concern. This four component solution accounted for 68% of the total variance with clear simple structure and item communalities ranging from .55 to .89. Intraclass correlations for these components were computed by comparing the component scores for each of an individual's three sampled areas of concern. The magnitude of ICCs varied by factor: For example, component 1, the belief that nothing would help and one had to learn to live with the problem, and component 3, the desire to get things back on track independently and to need to obtain more information, yielded high correlations of .40 and .45, respectively. This indicates that these two dimensions tended to be similar across all of an individual's areas of concern. Alternatively, component 4, the belief that a problem was not important enough to seek help, had an ICC of .16, indicating that this component was much more problem-specific. Component 2, feeling embarrassed, uncomfortable and judged about seeking care, had an intermediate ICC of .24.

**Table 5:** Item Endorsement and Principal Components Analysis of Influences on Decisions to Seek Care (N = 969 events in care from 387 clients)

**Items**	Response Rate*	**1**	**2**	**3**	**4**
Were afraid that nothing would help	25%	**0.73**	0.30	0.19	0.04
Felt like you should just learn to live with this concern	33%	**0.71**	0.01	0.39	0.13
Thought that getting help would lead to unwanted changes in your care	20%	**0.68**	0.29	0.04	0.25
Worried that getting help might make this problem worse	13%	**0.66**	0.19	0.09	0.03
Felt embarrassed or uncomfortable	22%	0.25	**0.85**	0.15	0.11
Thought that you might be judged	19%	0.25	**0.85**	0.15	0.09
Wanted to get things back on track on your own	53%	0.12	0.07	**0.84**	0.24
Needed more information about help that was available	49%	0.28	0.29	**0.72**	-0.10
Thought that this was not important enough to go for help	10%	0.18	0.14	0.12	**0.94**

*Proportion of events for which respondents indicated that this item influenced their decision to seek care "somewhat" or "a great deal".

Component Labels

1. Belief that Nothing Would Help – Had to Learn to Live With Problem

2. Felt Uncomfortable, Judged, Embarrassed about Seeking Care

3. Wanted to address things on your own – Needed more Information

4. Not Important Enough to Go For Help

Correlation analyses indicated that these components explained differences in help seeking. In particular, the decision to not consider professional help was significantly associated with lack of desire for additional information to get things on track (r = -.19, p < .001), the belief that it was not necessary to learn to live with the problem at hand (r = -.12, p < .04), and the feeling that the problem was unimportant (r = .10, p < .06). Alternately, the desire to obtain information to get things back on track was positively associated with both considering help (r = .16, p < .002) and seeking help (r = .11, p < .04). As might be expected, people already in care were less likely to feel that their concerns were not important enough to warrant formal help (r = -.13, p < .02).

Results of regression analyses shown in Table 6 indicate that these components differed across areas of concern and were associated with health care, health status, and demographic variables. Four patterns in help seeking become evident by examining results across predictor domains. The first major pattern in these results was that sicker people were more likely to view help seeking as futile. The belief that nothing would help was associated with greater use of supportive services (nursing, physical or occupational therapy, home assistance) and poorer health status (detectable viral load, lower quality of life). This view was also more common among respondents who had been HIV+ for a longer time. Conversely, patients who changed their care arrangements by enrolling in one of the new Medicaid Special Needs Plan were less likely to believe that nothing could help their problems.

**Table 6:** Regression Analyses – Barriers and Delays Encountered while Seeking Help

**Believed Nothing Would Help – Had to Learn to Live With Problem**		0.12	p < .001
Sexual Health and Risk Behavior (0.17)	Nursing Care (0.14)	Years Since HIV Diagnosis (0.12)	Spanish Speaking (0.26)
	Physical, Occupational Therapy, Rehab (0.14)	Undetectable Viral Load (-0.11)	Gay or Bisexual Identity (0.13)
	Home Assistance (0.13)	Psychological QOL (-0.11)	
	Special Needs Plan (SNP) Enrollee (-0.11)		

**Felt Uncomfortable, Judged, Embarrassed about Seeking Care**		0.13	p < .001

Psychiatric Symptoms (0.24)	Total Number of Providers (0.10)	Number of Lifetime Diagnoses (0.10)	Number of Sources of Income (-0.16)
Family Planning and Birth Control (0.10)	Special Needs Plan (SNP) Enrollee (-0.14)	Years Since HIV Diagnosis (-0.08)	
		Number of Symptoms (0.14)	

**Wanted to address things on your own – Needed more Information**		0.08	p < .001

Sexual Health and Risk Behavior (0.16)	Nursing Care (0.16)	Recent Hard Drug Use (0.12)	Latino Identity (0.20)
Substance Use (0.11)			Number of Sources of Income (-0.11)

**Not Important Enough to Go For Help**		0.1	p < .001

Substance Use (0.10)	Dental Care (-0.16)	Undetectable Viral Load (0.11)	Lives with Minor Children (0.09)
	Nursing Care (0.09)	Years Since HIV Diagnosis (0.10)	
		Recent Hard Drug Use (0.15)	
		Recent Unprotected Sex with HIV- Partner (0.14)	

**Difficulty getting an appointment with provider**		0.12	p < .001

Medical Problems and Symptoms (0.18)	Home Assistance (0.14)	Years Since HIV Diagnosis (0.17)	Spanish Speaking (0.11)
Psychiatric Symptoms (0.15)	Nursing Care (0.11)	Psychological QOL (-0.18)	Unstable Housing Situation (-0.10)
Specialty and Inpatient Care (0.15)			

**Language difficulties**		0.23	p < .001

Sexual Health and Risk Behavior (0.24)	Physical, Occupational Therapy, Rehab (0.21)	Psychological QOL (-0.18)	Spanish Speaking (0.33)
Specialty and Inpatient Care (0.12)	Nursing Care (0.19)	Recent Unprotected Sex (0.20)	Level of Education (0.12)
	Number of Providers Seen per Concern (0.12)		
	Special Needs Plan (SNP) Enrollee (-0.07)		

**Transportation problems or distance**		0.17	p < .001

Psychiatric Symptoms (0.22)	Home Assistance (0.11)	Undetectable Viral Load (-0.11)	African American (-0.14)
Medical Problems and Symptoms (0.19)		Involved in Sex Work (-0.13)	Level of Education (0.13)
Family Planning and Birth Control (0.13)		Psychological QOL (-0.15)	Number of Sources of Income (-0.13)
Dealing with Life Circumstances (0.18)		Recent Unprotected Sex (0.11)	Lives with Main Partner (0.14)

**Accessibility problems because of things like stairs, curbs, or lack of ramps**		0.12	p < .001

Dealing with Life Circumstances (0.17)	Number of Case Managers (0.12)	Physical QOL (-0.10)	Lives with Main Partner (0.10)
Sexual Health and Risk Behavior (0.16)	Psychiatric or Mental Health Care (-0.11)		
Adherence to Medical Instructions (0.15)	Number of Providers Seen per Concern (0.12)		
Specialty and Inpatient Care (0.13)			

**Inconvenient hours**		0.16	p < .001

Specialty and Inpatient Care (0.24)	Physical, Occupational Therapy, Rehab (0.21)	Number of Lifetime Diagnoses (0.11)	African American (-0.10)
General Wellness and Prevention (0.23)	Number of Referrals (0.12)	Psychological QOL (-0.12)	Monthly Income (-0.17)
Dealing with Life Circumstances (0.19)			
Adherence to Medical Instructions (0.16)			

**Cost of care or lack of coverage**		0.08	p < .001

Sexual Health and Risk Behavior (0.14)	Number of Case Managers (-0.13)		Number of Sources of Income (-0.14)
Psychiatric Symptoms (0.14)	Physical, Occupational Therapy, Rehab (0.09)		High School Graduate (0.14)

**Provider refusal to provide care**		0.05	p < .002

Family Planning and Birth Control (0.13)	Number of Referrals (-0.09)	Years Since HIV Diagnosis (-0.10)	
Sexual Health and Risk Behavior (0.13)		Number of Symptoms (0.09)	

**Delays in Care Made Problems Worse (Average)**		0.13	p < .001

Dealing with Life Circumstances (0.22)	Dental Care (-0.10)	Involved in Sex Work (0.15)	
Adherence to Medical Instructions (0.15)	Number of Case Managers (0.09)	Psychological QOL (-0.12)	
Specialty and Inpatient Care (0.13)	Number of Providers Seen per Concern (-0.11)	Recent Hard Drug Use (0.10)	
		Current Tobacco Smoker (0.11)	

The second pattern, feelings of being embarrassed and judged when seeking help were associated with mental health and family planning concerns, both potentially stigmatizing issues in this population. Findings suggest that this feeling may be predominant among people diagnosed at a later stage of disease: correlates included having a higher number of providers, more symptoms, and more lifetime diagnoses, despite fewer years since diagnosis. These individuals also tended to have fewer sources of income. Again, people who were willing to enroll in a Special Needs Plan were less likely to report that they felt uncomfortable seeking help.

The third pattern, people involved in riskier behavior were more inclined to avoid professional care. Deterrents to help seeking were associated with two of the least prevalent areas of concern, sexual risk and substance use. The beliefs that nothing would help, that the concern was not important enough to seek help, and that it was preferable to address problems independently may account for the lower reporting of concerns in these areas. Respondents who reported higher sexual risk behavior and substance use at baseline were also more inclined to dismiss professional help and to want to handle things on their own. Again, having an undetectable viral load contributed to the feeling that concerns did not warrant professional help.

Finally, deterrents to help seeking reflected several social and cultural differences evident in our sample. Spanish-speaking respondents were likely to have a fatalistic view of help-seeking as futile. Gay or bisexual respondents also expressed this feeling of futility. Hispanics in general were more likely to want more information in order to resolve concerns on their own. Clients with fewer sources of income were more likely to feel stigmatized, and to try to address problems on their own. Individuals with minor children in the household were likely to see their own needs as unimportant. These findings highlight the ability of the Dynamics of Care assessment to identify help-seeking issues and concerns specific to particular individuals and particular needs.

### Barriers and delays encountered while seeking help

Of the 387 clients who reported at least one event in care during the preceding three months, 92% (355) were either seeking or receiving services for at least one of the concerns that they had identified. For these respondents, we were able to ask about barriers to care that they had encountered while seeking care, and whether these barriers contributed to delays in care and actually obtaining care. We included questions about the following barriers: difficulty getting an appointment (encountered by 14% of respondents); problems with transportation or distance (13%); inconvenient hours (12%); accessibility problems due to stairs, curbs, lack of ramps and the like (8%); language difficulties (5%); cost of care or lack of coverage (4%); and provider refusal to provide care (1%). Overall, 72% of clients reported none of these barriers, 13% had encountered one barrier, 8% reported two barriers, 4% three barriers, and 4% four or more. Intraclass correlation coefficients were quite high for most of these barriers, including transportation (.69), inaccessibility (.58), language problems (.43), costs (.39) and hours (.35), indicating that people were generally dealing with the same barriers across several domains of care. However, this finding did not hold for appointment difficulties (.18) and provider refusal (.00), which were apparently more provider-specific.

As is evident in Table 6, different barriers to care were associated with different areas of concern. For example, appointment difficulties were more of a problem for formal medical, specialty and psychiatric needs. Costs or lack of coverage were a factor in obtaining help for psychiatric and behavioral risk concerns. Although rare, perceived provider refusal to offer care was encountered in seeking help for family planning and sexual health. In general, people who had more providers and worse health status at baseline encountered more subsequent barriers. Spanish-speaking clients (in contrast with African-American clients) and clients with lower monthly incomes and fewer sources of income were all more likely to encounter one or more barriers to care. Interestingly, two client characteristics that might have been expected to facilitate access to care were associated with higher barriers: living with a main partner and having a higher level of education. Since having a partner was associated with transportation and accessibility problems, we examined whether living with a partner was a proxy variable for disability or for care giving responsibilities, but neither appeared to be the case. Education was associated with costs and coverage barriers; it is possible that these clients were experiencing issues with Medicaid spend down, or that they were exploring services that were not covered by Medicaid.

The barriers to care reported here were only weakly associated with actually receiving versus seeking care. The only significant barrier that kept clients from connecting to care was transportation (r = -.16, p < .003). However, many barriers contributed significantly to delays in care that made the problem worse. Such delays were reported by 23% of respondents, and were associated with inconvenient hours (r = .23, p < .001), provider refusal (r = .21, p < .002), difficulty getting appointments (r = .20, p < .001), inaccessibility (r = .17, p < .001), transportation (r = .17, p < .001), and costs of care or insurance coverage (r = .13, p < .02). Combined, these barriers explained 10% of the variance in delays in care (p < .001). Additional correlates of delay are presented on Table 6. Delays were worse for concerns involving life circumstances, adherence, and specialty care. Clients who experienced delays had fewer providers in their baseline network, particularly dentists. However, they were more likely to be working with case managers at baseline. Delays in care were especially associated with risk behaviors at baseline, including sex work, substance use and smoking, suggesting that these behaviors might make it more difficult for clients to initiate care.

### Perceived quality of interaction with main providers

Of the 355 respondents who had sought help for at least one of their concerns, 87% (308) connected with care and were able to discuss services that they had received over the past three months. In general, satisfaction with care was quite high. Of the 52% of sampled events (500 out of 969) where contact with a main provider occurred, clients were "very satisfied" with the help they received 88% of the time, "somewhat satisfied" 8% of the time, "somewhat dissatisfied" 2% and "very dissatisfied" 2%. In order to obtain a satisfaction variable with a better distribution, we determined the lowest level of satisfaction reported by clients for any of their sampled areas of care. The majority of clients, 82%, were very satisfied with the care they received across all sampled areas. Of the remainder, 11% rated themselves as somewhat satisfied with at least one episode of care, 4% were somewhat dissatisfied with at least one area, and 3% were very dissatisfied with the help they received for at least one sampled event. Intraclass correlation of satisfaction ratings was .17, indicating only a modest association. In other words, if a patient was dissatisfied with help received for one area, they were slightly more likely to report dissatisfaction in other areas. Note that clients' lowest rating of satisfaction with help received was correlated with the more global rating of satisfaction with available services used to select events for probing (r = .35, p < .001), although this correlation was necessarily attenuated by the skewed distribution of this measure. Consistent with the literature on satisfaction assessment, regression analysis presented on Table 7 show greater dissatisfaction among clients receiving assistance with medical problems and specialty care, and among individuals who had more health concerns at baseline, including lower CD4 count, more diagnoses, and more symptoms. However, current cigarette smokers reported higher satisfaction with care.

**Table 7:** Regression Analyses – Perceived Quality of Interaction with Main Provider

**Lowest Satisfaction with Care from Main Provider for this Concern**		0.08	p < .001
Medical Problems and Symptoms (-0.17)		CD4 Count Greater than 200 (-0.11)	
Specialty and Inpatient Care (-0.17)		Number of Lifetime Diagnoses (-0.11)	
		Number of Symptoms (-0.12)	
		Current Tobacco Smoker (0.15)	

**Provider did not address problem**		0.2	p < .001

Dealing with Life Circumstances (0.25)	Special Needs Plan (SNP) Enrollee (-0.16)	Undetectable Viral Load (-0.15)	Number of Sources of Income (-0.14)
Medical Problems and Symptoms (0.22)		Psychological QOL (-0.18)	Lives with Minor Children (0.09)
General Wellness and Prevention (0.25)		Recent Unprotected Sex (0.09)	
Family Planning and Birth Control (0.15)			

**Provider expressed disrespect or blame, patient lacks confidence in care**		0.12	p < .001

Adherence to Medical Instructions (0.19)	Physical, Occupational Therapy, Rehab (0.13)	Number of Lifetime Diagnoses (0.10)	Gay or Bisexual Identity (0.10)
Specialty and Inpatient Care (0.12)		Having a Current Partner who is an IDU (-0.08)	Criminal Justice Involvement (0.12)
		Physical QOL (-0.14)	
		Current Tobacco Smoker (-0.13)	

**Unclear instructions or impractical help**		0.11	p < .001

Specialty and Inpatient Care (0.19)		Number of Lifetime Diagnoses (0.10)	African American (-0.11)
		Psychological QOL (-0.15)	Any Employment (0.09)
		Alcohol Use (-0.10)	
		Recent Unprotected Sex (0.11)	

**Family disagreement about care, care interferes with responsibilities**		0.23	p < .001

Psychiatric Symptoms (0.27)	Physical, Occupational Therapy, Rehab (0.21)	Number of Lifetime Diagnoses (0.15)	Monthly Income (-0.10)
Dealing with Life Circumstances (0.23)	Number of Referrals (0.12)	Involved in Sex Work (-0.07)	
Specialty and Inpatient Care (0.19)		Number of Symptoms (0.21)	
Sexual Health and Risk Behavior (0.18)		Recent Unprotected Sex (0.19)	
Substance Use (0.17)		Current Tobacco Smoker (-0.11)	

In addition to satisfaction ratings, we also asked clients to rate whether they encountered specific difficulties in their care. These 14 items and their orthogonal varimax principal component structure after promax rotation are presented in Table 8. This four component solution accounted for 62% of the total variance with adequate simple structure and item communalities ranging from .52 to .83, except for one item, "taking care of this problem interfered with my other responsibilities" which had a communality of .33. We again examined intraclass correlations to determine whether similar components were correlated across individuals' three sampled areas of care. ICC varied substantially across these four different components, consistent with their interpretation. Component 1, the provider did not respond to the client's questions or address the problem, had an ICC = .17, suggesting that variance was primarily due to specific events rather than patient differences. Similarly, component 3, unclear instructions or impractical help, had an ICC = -.115. This makes sense given the inherent specificity of particular instructions. Component 2, the sense that a provider expressed blame and disrespect, was more consistent across areas with ICC = .28, perhaps reflecting patients who have a pervasive feeling of alienation or mistrust of the health care system. Component 4 involves family disagreement and interference with responsibilities, which could easily carry over into several areas of care, had a relatively high ICC = .35.

**Table 8:** Item Endorsement and Principal Components Analysis of Difficulties Encountered in Care (N = 500 Events in Care from 308 Clients)

**Items**	Response Rate*	**1**	**2**	**3**	**4**
Your problem was not taken seriously	5%	**0.87**	0.12	0.08	-0.07
The provider did not really get to the real problem	5%	**0.78**	0.09	0.21	0.17
The provider did not understand your concerns	5%	**0.69**	0.13	0.30	0.17
You had questions were not answered	5%	**0.68**	0.08	0.11	0.21
The provider expected you to do too much on your own	9%	**0.66**	0.37	0.04	0.13
There was not enough time to discuss the problem fully	7%	**0.50**	-0.03	0.26	**0.47**
You felt like you were being judged or blamed	5%	0.24	**0.76**	-0.15	0.09
You experienced disrespect or discrimination	1%	-0.10	**0.74**	0.10	0.19
You did not have confidence in the care you received	6%	0.28	**0.58**	0.38	-0.02
You disagreed with what the provider told you to do	10%	0.38	***0.50***	0.25	0.25
Instructions were unclear or hard to understand	4%	0.18	0.00	**0.88**	0.14
The help offered was not practical	4%	**0.44**	0.33	**0.57**	-0.04
Family or significant others disagreed with the provider	5%	0.07	0.12	-0.05	**0.87**
Taking care of this problem interfered with your other responsibilities	10%	0.18	0.26	0.13	**0.47**

*Proportion of events for which respondents indicated that this item influenced their decision to seek care for any of their concerns.

Component Labels

1. Provider did not respond to questions or address problem

2. Provider expressed disrespect, blame, patient lacks confidence in care

3. Unclear instructions or impractical help

4. Family disagrees about care, care interferes with responsibilities

Difficulties in care were associated with different areas of concern and baseline health status, as presented on Table 7. Clients concerned about life circumstances, medical problems, wellness, and family planning were more likely to say that a provider did not address their problems. This difficulty was also associated with a broad range of client needs and vulnerabilities, including higher viral load, poorer psychological well-being, greater sexual risk behavior, fewer sources of income and the presence of minor children in the household. Clients who had enrolled in a Special Needs Plan were less likely to experience difficulties getting their problems addressed and questions answered.

More serious problems in relationships with providers, captured on the second component, were associated with medication adherence and specialty care. Clients receiving physical or occupational therapy were more likely to mention these difficulties, as were people who had more lifetime diagnoses, had greater physical quality of life problems, were gay or bisexual, or were involved in the criminal justice system. Several correlations suggest that people currently involved in riskier behavior (smokers, partners of injection drug users) were less likely to attribute disrespect or blame to their providers. Recipients of specialty care as well as those with more lifetime diagnoses, poorer psychological quality of life and higher sexual risk behavior were more likely to report that they had difficulty with instructions and that the help they were offered was impractical. It is also noteworthy that clients who were employed were more likely to report that help was impractical. Family disagreements about care and interference of care with other responsibilities were most strongly associated with concerns about psychiatric symptoms and life circumstances, but were also related to substance use, sexual risk and specialty care concerns. Disagreements were associated with a greater number of medical referrals, as well as poorer physical health, greater risk behavior and lower monthly income. Again people who used tobacco or who engaged in sex work were less likely report these disagreements.

Greater difficulties in care were negatively correlated with satisfaction with help received, including unclear instructions (r = -.57, p < .001), provider not responding to questions (r = -.53, p < .001), perceived disrespect (r = -.42, p < .001), and family disagreements with care (r = -.30, p < .001). Overall, difficulties accounted for 42% of the variance in satisfaction (p < .001).

### Problem resolution

Given its focus on specific events in care, it is natural to ask about problem resolution as part of the Dynamics of Care interview. This question was pertinent to all events, even if respondents had not started to seek or receive care. Of the 969 events in care sampled, 17% were rated as "all settled," 16% as "mostly settled," 28% as "partly settled," and 39% as "not at all settled." Problem resolution had a moderate ICC of .21 across different areas of concern, indicating that these ratings were largely event-specific. Across participants, only 7% of respondents stated that all of the events and concerns identified through screening were fully resolved at the time of the Dynamics in Care interview, and 66% said that none of the sampled concerns were fully resolved. Given the wide range of variation across different areas of concern, we created a problem resolution index that yielded a weighted average score across areas sampled: individuals received 1 point for each concern that was fully resolved, 1/2 point for concerns that were mostly resolved, -1/2 point for concerns that were partly resolved and -1 point for concerns that were not at all resolved. This weighted index made it possible to examine factors that influenced overall problem resolution. These results are presented on Table 9.

**Table 9:** Regression Analyses – Problem Resolution

**Problem Resolution Index**		0.11	p < .001
General Wellness and Prevention (-0.16)	Number of Providers Seen per Concern (-0.09)	Years Since HIV Diagnosis (-0.09)	Gender (Female) (0.09)
Psychiatric Symptoms (-0.16)	Special Needs Plan (SNP) Enrollee (0.10)	Involved in Sex Work (-0.08)	Unstable Housing Situation (-0.10)
Specialty and Inpatient Care (-0.15)		Psychological QOL (0.15)	

Problem resolution was lower among individuals whose concerns involved psychiatric symptoms, wellness and prevention, and specialty or inpatient hospital care. Individuals who sought care from several providers for particular concerns had a lower problem resolution index. However, individuals who enrolled in a Special Needs Plan were likely to report better problem resolution, perhaps reflecting their proactive choice in care. Better problem resolution was associated with fewer years living with HIV and better psychological quality of life at baseline. Individuals who had a history of sex work or unstable housing reported worse problem resolution. Problem resolution was also higher among women compared to men in our sample.

We also examined the impact of the Dynamics of Care on problem resolution. We conducted a series of analyses to examine how each section of the Dynamics of Care interview was related to the problem resolution index, including significant correlates and overall variance explained by each section. In terms of help seeking status, receiving help was associated with problem resolution (r = .22, p < .001), in contrast to seeking help (r = -.21, p < .001), and contemplating help (r = -.19, p < .001). People who were deterred from help seeking because they felt embarrassed also reported lower problem resolution (r = -.17, p < .001). However, higher problem resolution was associated with not considering help (r = .11, p < .02), and with the belief that a problem was not important enough to seek help (r = .15, p < .004). Overall, help seeking accounted for 14% of the variance in problem resolution (N = 387, p < .001).

Problem resolution was less directly related to the barriers that people encountered while seeking help. Inconvenient hours of service (r = -.18, p < .001) and transportation problems (r = -.11, p < .05) were both associated with diminished problem resolution, as were the experience of delays that made the problem worse (r = -.18, p < .001). Overall, barriers and delays accounted for 5% of the variance in the problem resolution index (N = 355, p < .001). Satisfaction and difficulties encountered in care also explained further variance in problem resolution. The correlation between satisfaction and problem resolution was (r =.16, p < .005). Difficulties encountered in care were each negatively correlated with problem resolution, including family disagreements and conflicting responsibilities that are affected by care (r = -.16, p < .005), lack of response to questions (r = -.13, p < .02), perceived disrespect and lack of confidence in care (r = -.13, p < .02) and unclear instructions (r = -.12, p < .04). All together, satisfaction and difficulties explained 4% of the variance in problem resolution (N = 308, p < .04). This is consistent with the observation in the literature that satisfaction alone does not adequately capture the degree of resolution of problems in care.

### Predictive validity of Dynamics of Care measures

In order to determine whether measures derived from the Dynamics of Care interview were useful in understanding health delivery outcomes, we conducted a series of regression analyses to predict different service use measures and quality of life at our six month follow-up, three months after the dynamics interviews were completed (N = 291). Follow-up variables included several aspects of service use in the month preceding the six month interview: number of provider office visits (mean is about 2 visits per month, with 19% reporting five or more visits), number of home visits (mean is between zero and one, with 8% reporting three or more visits), telephone contacts (mean is slightly less than one phone call per month with 13% reporting 3 or more contacts), and overnight stays in the hospital (5% had one or more overnight hospitalizations in the past month). We also examined how Dynamics in Care related to changes in quality of life over six months, from the baseline interview. About 30% of the sample declined and 46% of the sampled improved by 5 or more points (on a 100 point scale) in physical quality of life, while 33% declined and 44% improved by 5 or more points in psychological quality of life. In order to conduct a stringent test of the value of the dynamics of care measure, we controlled baseline quality of life scores prior to entering dynamic measures. Since we used absolute measures of follow-up service utilization rather than change scores, we controlled for baseline measures of years since diagnosis, symptoms, opportunistic infections and malignancies, CD4 count over 200, undetectable viral load, number of conditions for which treatment has not been obtained, and quality of life. We also controlled for the occurrence of events in care identified at three months, to distinguish emergent needs from processes in care. Partial correlation coefficients of dynamics of care predicting six month utilization and QOL are presented in Table 10.

**Table 10:** Partial Correlations of Utilization and Quality of Life at Six Months to Three Month Dynamics of Care Measures, Controlling Baseline Variables and Events in Care

	Office Visits	Home Care	Phone Calls	Hosp. Stays	MOS Phys	MOS Psych
**Help Seeking Status at 3 Months**						
Already Receiving Help			.15			
Considering Help						
Would not Consider Help			-.12	.14		
**Help-Seeking Influences**						
Felt Uncomfortable, Judged, Embarrassed about Seeking Care	.15					
**Barriers to Care**						
Accessibility Problems	.13		.15		-.15	
Inconvenient Hours		-.12				
Provider Refusal	.17					
**Satisfaction and Difficulties in Care at 3 Months**						
Satisfaction with Provider			-.13	-.17		
Provider did not respond to questions or address problem		-.19	.22		-.14	
Unclear instructions or impractical help			.15			
Family disagrees about care, care interferes with responsibilities		-.14				-.17

As Table 10 demonstrates, both service use and QOL indicators had small but significant incremental relationships with the Dynamics in Care measure. A greater number of visits to providers at six month follow-up was associated with feeling embarrassed, having problems with access, and encountering provider refusals at three months. Apparently respondents were able to overcome these concerns and increase service use. These results are also consistent with the interpretation that earlier delays can lead to greater use at a later time. After controlling for baseline and other health status indicators, receipt of home-based care was associated with better access to providers at three months including better hours, responses to questions, and fewer familial disagreements about care. The frequency of telephone contacts with providers at six months was related to physical difficulties in getting access, difficulty with getting questions answered, unclear instructions, and lower satisfaction. Although hospitalizations were infrequent, it is noteworthy that dissatisfaction with provider and dismissal of professional help at three months were significant predictors of hospitalizations at six months. After controlling for baseline levels, decline in physical quality of life was associated with problems with accessibility and provider responsiveness, while diminished psychological quality of life was related to family-provider disagreements and interference with responsibilities.

## Discussion

The Dynamics of Care assessment was designed to be a flexible, integrated approach to determine individuals' salient service needs, help seeking, and experiences in care. Procedures for screening events in care were successful in capturing current concerns of Medicaid clients. It is noteworthy that nearly half of these events had not yet been brought to the attention of care providers. One significant advantage of patient-oriented assessment is the ability to gain perspectives on care that are not available from any other source. This assessment procedure sheds light on concerns that may not make it into care because of patients' sense of futility, embarrassment, or independence.

The Dynamics of Care approach also permits contrasts between different concerns in care. Although the extent of cross-situational consistency represented by ICCs differed from variable to variable, this variation appeared to make sense in terms of the construct under consideration. As our analyses show, help seeking decisions and satisfaction with care each appear to be situation-specific, rather than person-specific. In other words, individuals may choose to seek help for one problem but avoid assistance for another. Alternatively, barriers to care appear to be more cross-situational for respondents in this sample, and may be associated with clients' living situation or care arrangements. However, this seemed to depend upon the nature of the barrier.

Two inventories embedded in this assessment, to assess potential deterrents to help seeking and difficulties encountered in care, demonstrated very clear principal component structure, and were helpful in understanding aspects of help seeking and satisfaction with care. The problem resolution index also proved to be independent from satisfaction with care and had better distributional properties than satisfaction. The Dynamics of Care measure has clear potential to distinguish situations in which one outcome is achieved in lieu of the other.

The Dynamics of Care assessment was associated with subsequent utilization and change in quality of life. Although these effects were small after covariates were taken into account, the nature of the relationships made sense. For example, people with more questions and unclear instructions at three months said that they spent more time on the phone with providers at six months. Home care was less likely when clients reported family disagreements about care. Although certain dynamics variables were correlated with baseline psychological quality of life, they were still associated with changes in service use and physical quality of life after controlling for this baseline measure.

Despite these strengths, the Dynamics of Care procedure has several limitations that must be highlighted. The Dynamics of Care data are somewhat complicated to work with, including a different number of concerns sampled per respondent and different skip patterns dependent upon help-seeking status. The number of events sampled was also limited by available time. Additional information might be gained from probing more events, and it is necessary to establish that the client's "biggest concern" really is the most important one to discuss.

It is also important to distinguish the algorithm used to assess service use from the particular instrument discussed here. The particular events sampled were very much driven by the questions and concerns we had regarding Medicaid-supported HIV/AIDS care and the Special Needs Plan. Other ways of identifying and sampling events in care could be developed. It would be possible to focus in more depth on one aspect of care (for example, by unpacking the "life circumstances" category). It would also be possible to use different strategies to sample events, including standard symptom-related screeners, discharge planning assessments, and even provider-nominated categories. Assessment of help seeking decisions could include more items about positive reasons to obtain care as well as deterrents, and difficult items could be balanced with benefits of care. Nonetheless, all of these modifications would fit readily into a Dynamics of Care framework that emphasizes the analysis of a specific concern, problem, or need. We view this as a general framework that must be tuned to the specific assessment situation, much like social network analysis.

## Conclusion

In this report, we provided a psychometric overview of the Dynamics of Care assessment and we constructed global summary variables for this purpose. However, we have used or plan to use Dynamics of Care data in a number of different ways:

• To structure and amplify case reports on individual clients

• To highlight and monitor specific quality improvement projects

• To determine whether primary care providers respond differently to different needs

• To examine interaction effects among areas of concern and patient vulnerability measures – to see whether patients' known behavioral health risks play out as problems in care.

• In addition, we have also developed a follow-up reassessment of the Dynamics of Care measure to track the trajectory of specific problems over time. For example, how long do individuals strive to overcome barriers and at what point do they say that help seeking is futile?

This focus on specific problems in context is intended to provide more sensitive and informative ways to understand processes in care from the patient's perspective. This approach leads to a more grounded and systematic description of health care that can be used to direct new programs and resources to the patients and situations that require them.

## Abbreviations

DoC stands for Dynamics of Care. SNP stands for Special Needs Plan. The study was designed to evaluate New York State's Special Needs Plan – a comprehensive care model created for people with HIV and their children that was developed as an alternative to both Medicaid fee-for-service (FFS) and to Medicaid managed care. MCO stands for Managed Care Organization. HRSA stands for Health Resources and Services Administration. ARS stands for Acquiescent Response Set. NYSDOH stands for New York State Department of Health. DAC stands for Designated AIDS centers. ECSQ stands for Events in Care Screening Questionnaire. QOL stands for Quality of Life. GED stands for General Equivalency Diploma. PWHA stands for People living with HIV/AIDS. ICC stands for Interclass correlations

## Competing interests

The author(s) declare that they have no competing interests.

## Authors' contributions

Bruce Rapkin (BR) is the Principal Investigator of the project; he performed the statistical analysis and wrote the analysis and discussion sections. Elisa Weiss (EW) is the Project Director and took the lead in writing the background and methods sections. Rosy Chhabra (RC) is the former Project Director and contributed to the development of the measures and study design. Laura Ryniker (LR) is the Project Coordinator and helped to write the manuscript. Shilpa Patel (SP) participated in the data collection of the study and helped to write the manuscript. Jason Carness (JC) participated in the data collection of the study and helped to draft the background section. Roberto Adsuar (RA) participated in the data collection of the study and helped to draft the manuscript. Wendy Kahalas (WK) participated in the statistical analyses for the paper and reviewed many versions of the analysis section. Carol DeLaMarter (CD) participated in the design of the study and measures; she also reviewed and provided feedback on earlier versions of the manuscript. Ira Feldman (IF) participated in the design of the study and measures; he also reviewed and provided feedback on earlier versions of the manuscript. Judy DeLorenzo (JD) participated in the design of the study and measures; she also reviewed and provided feedback on earlier versions of the manuscript. Ellen Tanner (ET) participated in the design of the study and measures; she also reviewed and provided feedback on earlier versions of the manuscript.

All authors read and approved the final manuscript.
